# Association of clock gene variants with Autism Spectrum Disorder in South Indian population

**DOI:** 10.1186/1755-8166-7-S1-P117

**Published:** 2014-01-21

**Authors:** Ann Mary Alex, PA Suresh, Moinak Banerjee

**Affiliations:** 1 Human Molecular Genetics Laboratory, Rajiv Gandhi Centre for Biotechnology, Kerala, India; 2 Institute for Communicative and Cognitive Neuro Sciences, Kerala, India

## Background

Autism Spectrum disorder is a group of neurodevelopmental disorders that manifests in the first three years of life. Social impairments, communication difficulties and repetitive/stereotyped behaviour are the common symptoms of the spectrum. One of the major endophenotype associated with the disease is circadian and sensory dysfunction. Circadian dysfunction is mainly observed by difficulties in sleeping. Around 56-83% of patients with ASD suffer from sleep problems. There is an endogenous circadian clock that regulates the sleep and wakefulness, cognitive function, systematic hormonal release and body temperature. We hypothesize that the genes functioning to maintain this molecular clock may be associated with ASD either directly or by its transcriptional regulation of other genes.

## Materials and methods

The study population was from the Malayalam speaking population of Kerala. The patients were diagnosed and characterized based on DSM-IV criteria. We selected 2 genes which are core clock components, *hCLOCK* and *PER3* due to its functional relevance. The *CLOCK* gene is the first essential component of the mammalian clock and was found to be associated with circadian rhythm sleep disorders. *PER3* gene is implicated in delayed sleep phase syndrome and extreme diurnal preference. Single Nucleotide Polymorphisms in both genes were studied for association with the disease. Genotyping was done by sequencing and PCR RFLP.

## Results and conclusion

Genotypic and allelic frequencies of the SNPs studied were analyzed to understand if there exists an association with the disease. We could not find any association with the 8 polymorphisms screened in *hCLOCK* gene in our population. However we were able to get an association with a VNTR in *PER3* gene, with the 5 repeat allele being the risk allele. This is also the first report of *PER3* gene being associated with ASD.

